# Frontal white matter architecture predicts efficacy of deep brain stimulation in major depression

**DOI:** 10.1038/s41398-019-0540-4

**Published:** 2019-08-21

**Authors:** Volker A. Coenen, Thomas E. Schlaepfer, Bettina Bewernick, Hannah Kilian, Christoph P. Kaller, Horst Urbach, Meng Li, Marco Reisert

**Affiliations:** 1 Department of Stereotactic and Functional Neurosurgery, Freiburg University Medical Center, Freiburg, Germany; 2 Medical Faculty, Freiburg University, Freiburg, Germany; 3 Department of Neurosurgery, Bonn University Medical Center, Bonn, Germany; 4 BrainLinks/BrainTools, Cluster of Excellence, Freiburg University, Freiburg, Germany; 5 Neuromod, Center for Basics in NeuroModulation, Freiburg University, Freiburg, Germany; 6 Department of Interventional Biological Psychiatry, Freiburg University Medical Center, Freiburg, Germany; 7 Department of Psychiatry and Psychotherapy, Bonn University Medical Center, Bonn, Germany; 8 Department of Geronto-Psychiatry, Bonn University Medical Center, Bonn, Germany; 9 Department of Neuroradiology, Freiburg University Medical Center, Freiburg, Germany; 10 Division of Neuroradiology/Department of Radiology, Bonn University Medical Center, Bonn, Germany; 11 Clinical Affective Neuroimaging Laboratory, Department of Psychiatry and Psychotherapy, University of Tübingen, Tübingen, Germany

**Keywords:** Depression, Prognostic markers

## Abstract

Major depression is a frequent and severe disorder, with a combination of psycho- and pharmacotherapy most patients can be treated. However, ~20% of all patients suffering from major depressive disorder remain treatment resistant; a subgroup might be treated with deep brain stimulation (DBS). We present two trials of DBS to the superolateral medial forebrain bundle (slMFB DBS; FORESEE I and II). The goal was to identify informed features that allow to predict treatment response. Data from *N* = 24 patients were analyzed. Preoperative imaging including anatomical sequences (T1 and T2) and diffusion tensor imaging (DTI) magnetic resonance imaging sequences were used together with postoperative helical CT scans (for DBS electrode position). Pathway activation modeling (PAM) as well as preoperative structural imaging and morphometry was used to understand the response behavior of patients (MADRS). A left fronto-polar and partly orbitofrontal region was identified that showed increased volume in preoperative anatomical scans. Further statistical analysis shows that the volume of this “HUB-region” is predictive for later MADRS response from DBS. The HUB region connects to typical fiber pathways that have been addressed before in therapeutic DBS in major depression. Left frontal volume growth might indicate intrinsic activity upon disconnection form the main emotional network. The results are significant since for the first time we found an informed feature that might allow to identify and phenotype future responders for slMFB DBS. This is a clear step into the direction of personalized treatments.

## Introduction

Depression is a highly prevalent and disabling condition that is associated with high rates of morbidity and mortality. More than 300 million patients are affected worldwide^1^ and ~20–30% of these patients do not sufficiently respond to established treatments such as drug medication and/or psychotherapy^2^. There is preliminary evidence that some of the patients who suffer from treatment resistant depression (TRD) might respond to deep brain stimulation (DBS)^3,4^.

The most researched targets are the subgenual cingulate region (cg25, SCG = subgenual cingulate gyrus) and the ventral capsule ventral striatum (VC/VS)^5,6^. Despite efficacy in single center trials, replication in multicentric and controlled trials for these two pivotal target regions failed ^3,4^.

DBS of the superolateral medial forebrain bundle (slMFB) was proposed as a promising alternative for patients suffering from TRD^7^. Small case series showed promising effects^8–10^. The scientific basis for this target region is its DBS modulation as a superior regulator of the reward system with contact to most of the regions hitherto targeted with DBS^7^ and its widespread connections to reward-associated frontal lobe regions^11^ together with its direct influence on the ventral tegmental area (VTA) and the fact that anhedonia and hopelessness are the most prominent symptoms of major depression^12,13^. In our first two clinical case series 18 out of 24 TRD patients responded well to DBS of the slMFB. However, as 25% of patients did not benefit from slMFB DBS, identification of potential biomarkers is key to further improve patient selection and to optimize individually tailored DBS. From a whole range of putative biomarkers, functional analyses of networks involved in the processing of stimuli emotional valence seem very promising, because of their symptomatic involvement.

Modern approaches for the evaluation of DBS in movement disorder surgery typically use normal population connectomes together with achieved electrode positions and VAT modeling^14–16^. For the subgenual target region, which can be silent during surgical implantation and acute stimulation, connectivity analyses now augment the procedure, explain the effectiveness and might improve DBS outcome ^15–17^.

In contrast, here we examine the predictive power of preoperative morphometric and structural connectivity data to explain postoperative response variability in TRD patients with slMFB DBS, based on imaging and clinical response data from two clinical trials of slMFB DBS in TRD patients (ClinicalTrials. gov: NCT01778790 & NCT0109526). The goal is to identify informed features including electrode positions, analyses of VAT ( = volume of activated tissue) and connectivity as well as structural anatomical imaging that might allow for an explanation and prediction of clinical response.

## Methods and materials

### Participants

Analysis of 24 patients (9 female) receiving bilateral slMFB DBS (FORESEE & FORESEE II trials; ClinicalTrials. gov: NCT01778790 & NCT0109526). Experimental treatment according to tenets of the Declaration of Helsinki, reviewed by the IRB of Bonn University Medical Faculty. Written informed consent was obtained.

The detailed techniques of stereotactic and tractography assisted slMFB DBS implantation and stimulation have been published before^18^. Demographic details of the patients can be found in the supplementals. Response criterion for this study (and different from the clinical outcome criteria and published results): = / > 50% improvement in the Montgomery Asberg depression rating scale (MADRS) in 50% DBS-ON time. For more detailed clinical information about the considered cohort (including treatment courses and medication) we refer to refs. ^8,19^.

### Imaging acquisition

MR imaging data were acquired on a whole-body 3T MR system (Philips Healthcare, Best, The Netherlands) by using an 8-element phased-array head coil. The MR imaging examination comprised an isotropic T2-weighted fast spin-echo sequence, a DTI sequence, and 2 magnetization-prepared rapid gradient- echo scans. The parameters were the following: fast spin-echo: repetition time (TR) = 12.650 ms, echo time (TE) = 100 ms, field of view (FOV) = 254 mm, matrix = 176 × 176, 120 sections, sections thickness = 1.44 mm, and acquisition time = 3 minutes and 44 s. The resulting data were reconstructed to isotropic (1.44 × 1.44 × 1.44)-mm^3^ voxels.

#### Diffusion tensor imaging sequence

Single-shot spin-echo echo planar imaging pulse sequence with TR = 13.188 ms, TE = 84 ms, FOV = 256 mm, matrix = 128 × 28, 70 sections, section thickness = 2 mm, number of gradient directions = 32, *b*-value = 1000 s/mm2, sensitivity encoding factor 2.9, acquisition time = 7 minutes 54 s with isotropic reconstructed (2 2 2) mm^3^ voxels.

#### Anatomical T1/T2 contrast

A T1-weighted 3-D magnetization-prepared rapid gradient-echo sequence was acquired before (structural information) and after (vessel visualization) contrast administration (gadolinium-diethylene-triamine pentaacetic acid) with a sensitivity encoding factor = 4, TR = 8.5 ms, TE = 3.8 ms, flip angle = 8, FOV = 256 mm, matrix = 256 × 256, 160 sections, section thickness = 2 mm, acquisition time = 4 min 17 s. It resulted in reconstructed isotropic (1 × 1 × 1) mm^3^ voxels. All images were taken in axial orientation.

#### Preoperative CT

Stereotactic computed tomography (CT) scans were acquired on a 16-row multidetector scanner (Brilliance 8000, Philips Healthcare, Best, The Netherlands) with a stereotactic frame. Parameters were as follows: tube voltage = 120 kV, tube current = 350 mA, collimation = 16 × 0.75 mm, tube rotation time = 1 s, pitch = 0.942, matrix = 512 × 512, section thickness = 1.5 mm, increment = 1.5 mm.

#### Postoperative CT

Helical CT (within 12 hours after surgery) used the following parameters: tube voltage = 120 kV, tube current = 350 mA, collimation = 16 × 0.75 mm, tube rotation time = 0.75 s, pitch = 0.688, matrix = 512 × 512, section thickness = 2 mm, increment = 1 mm.

#### Human connectome project

T1-weighted data from S500 release^20^, 2014 was used. Overall, 396 subjects with Adult Self-report DSM-IV Depressive score normalized <65 were selected.

### Image processing and voxel based morphometry

The anatomical T1 contrast was used as the reference, and CT, T2, dMRI were registered to T1 space using SPM12. The electrode positions were automatically detected by an in-house software and manually refined.

Anatomical T1 images were analyzed using the Computational Anatomy Toolbox (http://dbm.neuro.uni-jena.de/cat12/CAT12-Manual.pdf) using Statistical Parametric Mapping software (SPM12, http:// www.fil.ion.ucl.ac.uk/spm/software/spm12). The default settings were used, which are described in detail in the CAT12 manual. White and gray matter segmentations were normalized to the Montreal Neurological Institute (MNI) template.

During normalization the segmentation are modulated by scaling with the amount of volume changes due to spatial registration, so that the total amount of white/gray matter in the modulated image remains the same as it would be in the original image. After normalization white and gray volumes maps underwent a Gaussian smoothing (FWHM = 7 mm) and were resliced onto an isotropic grid of resolution 3 mm.

As a normative sample T1 images from the Human Connectome project (HCP) corpus underwent the same CAT12 pipeline and white/gray matter density maps were extracted.

### slMFB-based volume analysis

To understand the involvement of the slMFB, we investigated relative white/gray matter volume changes within (white matter) and in the vicinity of the slMFB (gray matter) with respect to the MADRS response scores. For this analysis the slMFB population template constructed in^11^ was adopted. For white matter analysis, the slMFB ROI was defined by all voxels for which more than 5% of the population had a significant amount of slMFB streamlines visited (see^11^). For gray matter analysis, a mask containing all gray matter matter voxels in the vicinity of the slMFB is constructed. Therefore, the slMFB white matter ROI was dilated by a kernel with a width of 6 mm and intersected with a mask for gray matter. All volume densities were computed relative to the total slMFB volume, which was defined as the sum of densities within the slMFB ROI.

### Whole-brain volume analysis

In a further explorative analysis whole-brain white and gray matter volumes were analyzed. As we were looking for small effects we adopted an preprocessing approach which is common in genetic analysis^21^. In this approach large variations within the group (usually attributed to ancestry) are additionally used in modeling. Instead of considering SNPs (single nucleotide polymorphisms) as explanatory variables as in Price et al. ^21^, here the local WM/GM volumes were used as the explanatory variables.

Following^21^ the directions of largest variations were determined by a Principal Component Analysis (PCA). We used a control group from HCP (ref. ^20^, see Participants) to determine these components. Therefore, the T1w images of the HCP corpus underwent CAT12 processing pipeline. Then, a PCA was performed over the whole corpus and the first 10 axes of variations^21^ were selected and regressed out of the patient group.

### Tractography and microstructural measures

We compared fractional anisotropy (FA) and mean diffusivity (mD) as microstructural dMRI-measures on a voxel level. Subject specific FA maps and mD were normalized using the normalization parameters derived by CAT12. Prior to normalization the maps were smoothed with a Gaussian kernel (FWHM = 6 mm). After normalization the maps were resliced on a 3 mm grid and compared with MADRS response. For tractography we mainly followed the global approach^22,23^ as used in Coenen et al.^11^. An additional accumulation strategy was used to provide more robust statistics.

#### Selection of fibers

To determine the fibers activated we used the common quasi-static approximation of Maxwell’s equation^24^. The cylindrical contacts of the electrode were approximated by point contacts, i.e. the Poisson equation was solved analytically for point-sources according to the bipolar programming of the electrode. As boundary conditions the electric currents measured were used. For example, for a stimulation with one negative contact located at position *r*_*b*_ and one positive contact at location *r*_*a*_ we use expression $V\left( r \right) = \frac{{I_a}}{{4\pi \sigma |r - r_a|^2}} - \frac{{I_b}}{{4\pi \sigma |r - r_b|^2}}$ for the voltage distribution, where *I* denote the measured currents. As activation threshold 100 mV/mm^2^ was used and an isotropic conductivity of *σ* = 0.1*S*/*m* was assumed. More precisely, if a streamline visits a voxel with direction/tangent (*t*_0_, *t*_1_, *t*_2_) and the second-order spatial derivative of the voltage distribution in direction of the tangent reached $|t_it_jd^2V/dr_idr_j| \,{>}\, 100\,{\mathrm{mV/mm}}^{\mathrm{2}}$ (the tensor$d^2V/dr_idr_j$was computed on a dense grid with resolution 0.25 mm), then the streamline was selected as activated^25^. Additionally to the above described selection method, where the selection depends on the traversal direction of the fibers, we followed a conventional modeling which neglects the direction of the streamlines. We used the method introduced by Mädler et al.^26^ with an activation threshold of 0.15 mV/mm. All fibers that visit the volume of activate tissue were selected as activated. The activated streamlines were further subdivided into five different sub-bundles by using the Desikan-Killiany atlas. For warping from group to native subject space the deformation fields obtained from CAT12 were used. The following prefrontal cortical parcels were used (nomenclature in analogy to Coenen et al. ^11^: lateral orbitofrontal, medial orbitofrontal, rostral middle/frontal, superior frontal (including frontal pole), and pars caudalis/triangularis/opercularis/orbitalis. Each of these prefrontal segments was taken as an additional selection criterion for the terminals of the activated streamlines. The so obtained streamline counts were used to regress the MADRS response (non-normalized and normalized with total streamline count).

### Normative connectome

As the dMRI data present in this study is of rather poor quality, the tractographic analysis was also conducted for a normative connectome. For construction of the normative connectome the healthy HCP subject group was used. The raw diffusion data (dMRI) was warped to MNI space (by the warps constructed with CAT12) and averaged over the group and tracked by the global tractography approach^22,23^. The reorientation of the dMRI data was based on the local Jacobian matrix^27^. A similar template connectome was also used in Coenen et al.^11^ for depiction of the slMFB. The electrodes were also warped to MNI space and used in the manner as described above for streamline activation. On the other hand, the normative connectome is used in Figs. 2 and 4 for visualization.

### Statistics

Multiple regression analysis was used to model the relationship between the explanatory variables (white/gray matter volumes and streamline counts) and continuous MADRS response. Age and onset of disease served as independent covariates in all statistical analysis. *T*-tests on the regression slope of explanatory variables were conducted to assess significance in the VBM and tractographic analyses. During tractographic analyses two subjects had to be excluded due to poor quality of the diffusion MRI data. Correction for multiple comparisons was applied using the parametric False Discovery Rate (FDR) at a level of 5%. Additional permutation tests were conducted to underpin the findings (5000 permutations of the *N* = 24 subjects were performed). All statistical analysis was performed with MATLAB r2018a, Mathworks.

### Post hoc analysis

In addition, a leave-one-out (LOO) regression analysis for the white matter region found (peak cluster at threshold *p* < 0.01 uncorrected) in slMFB analysis was conducted. The predictive volume is computed to be the mean volume density within the peak cluster. A three-dimensional (age, onset and volume) linear regression model to predict MADRS response is trained using N-1 subjects and applied for the remaining subject. Results of the LOO analysis are depicted in Fig. 3.

To understand the relationship of the found region with respect to the reward/depression system, the found peak cluster is used to select streamlines in an HCP group connectome (same HCP connectome as used in Coenen et al.^11^) and visualized in Fig. 4. In addition, for better understanding, the selected streamlines are grouped by ROIs into different sub-bundles: anterior thalamic radiation (ATR), superolateral medial forebrain bundle (slMFB), forceps minor (FMIN), cingulum (CG), uncinate fascicle (UNC), inferior fronto-occipital fascicle (IFOF), and superior anterior fascicle (SAF). Finally, in Fig. 5 we compare the regions addressed by the peak cluster to the typical depression related DBS target in the subcallosal cingulate gyrus (MNI: 6, 22, −7.5, selection radius *r* = 3 mm) according to Riva-Posse et al.^15^.

## Results

**Fig. 1:**
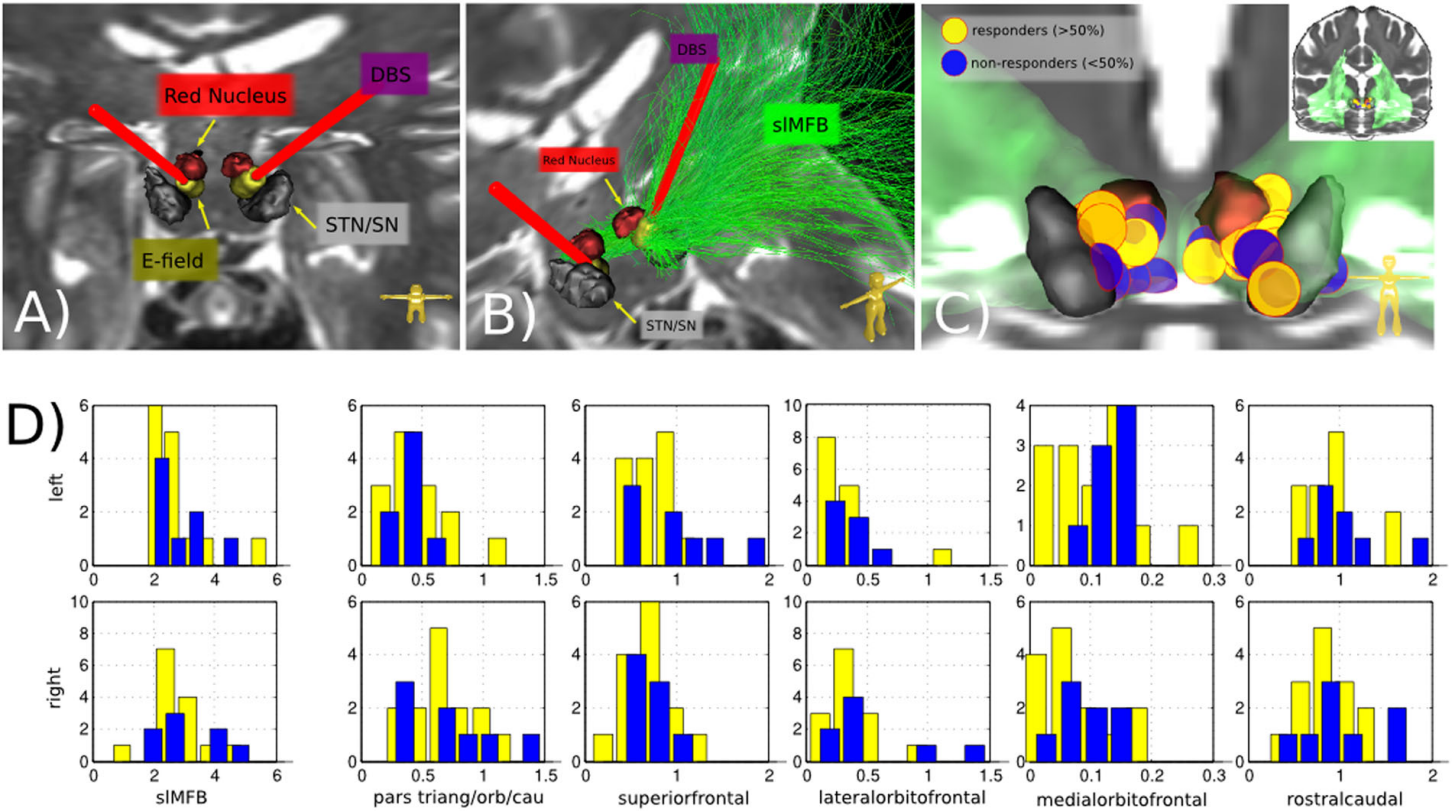
Analysis of activated bundles. **a**, **b** typical electrode positions. DBS electrodes (red, bilateral) with tips intercalated between STN/SNR and red nucleus (RN). Bipolar stimulation (VAT, yellow). **b**, slMFB (green) shown as streamlines only on left. **c** position of VATs (entire group, *N* = 24; yellow, responders; blue, non-responders). **d** Number of fibers addressed (in permille compared to the whole-brain connectome)

First, we used visual inspection and quantitative dMRI tractography to ascertain penetration of the target site by DBS electrodes (cf. Fig. 1a, b). Whole-brain reconstruction of individual connectomes showed that in all cases except one, about 0.2–0.5% of streamlines traversed the volume of activated tissue (for both activation models explored) around the stimulation electrodes (Fig. 1c). The projections of these activated streamlines were well associated with slMFB. However, no significant relationship between the treatment response and the strength or location of DBS induced activation, or frontal connectivity patterns was found (Fig. 1d). An analysis based on a normative connectome showed a very similar pattern. Also, no significant relationships between microstructural measures (FA and mD) and treatment response was found.

**Fig. 2:**
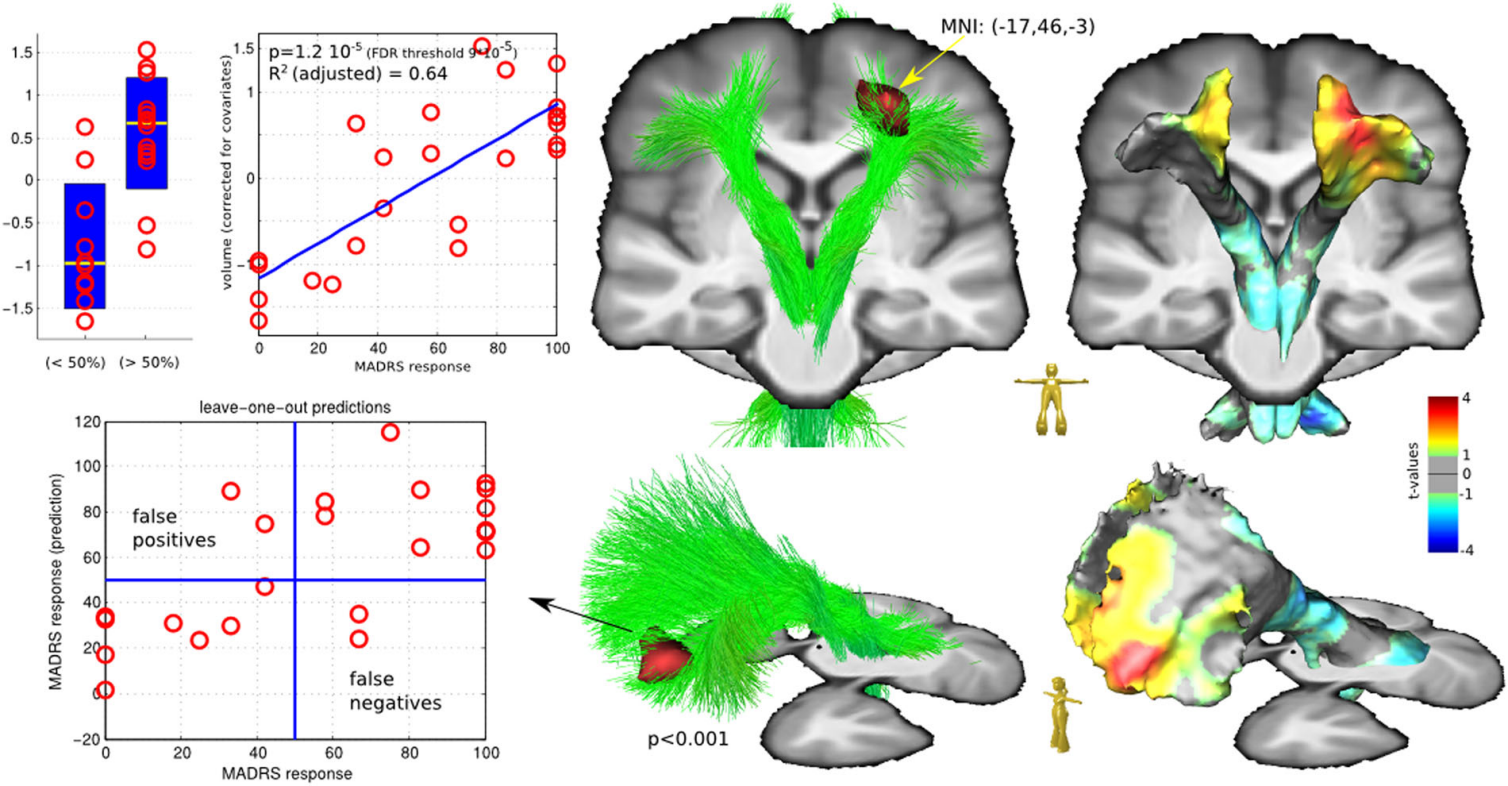
Correlation results and leave-on-out regression analysis of the slMFB region analysis. The fronto orbital region that is reached by left slMFB is significantly enlarged for responders, MNI coordinates (−17, 46, −3) at peak. Also right frontal enlargements and symmetric posterior shrinkage is observable (*p* < 0.01), but not significant at 5% FDR

We further studied the morphometry of white and gray matter associated with the slMFB and found significant positive relationship between treatment response and enlargement of the white matter in left fronto-polar slMFB terminals (significant at 5% FDR with *p* < 0.0001). Notably, this enlargement was confined to white matter and not to associated cortical regions (Fig. 2).

**Fig. 3:**
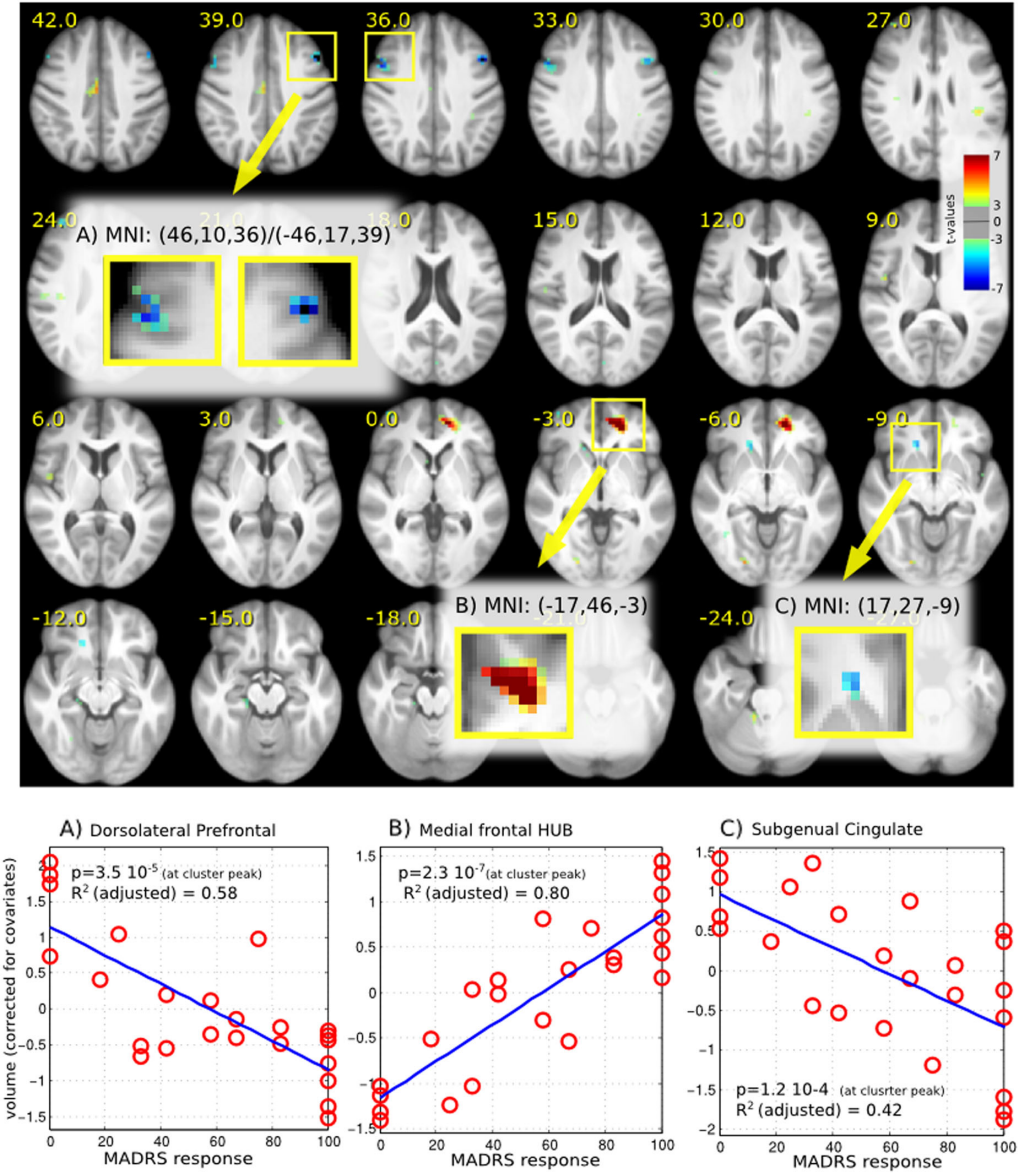
Results of whole-brain analysis support the finding from the region-based analysis. Additional to the fronto orbital region (**b**) two other regions showed a relationship (**a**, **c**), but not significant after FDR correction

In order to assess the anatomical specificity of the white-matter enlargement in left fronto-polar slMFB for successful DBS in TRD patients, we repeated the analysis on the whole-brain level with PCA correction. In this analysis (see Fig. 3 for presentation of results) the region was also found to be highly significant (*p* < 10^–6^, *R*^2^ adjusted = 0.8). In addition, we found two other regions, which are associated with the reward system but are inversely correlated with MADRS response: one in the dorsolateral frontal region (DLPFC, MNI 46, 10, 36/−46, 17, 39 with *p* < 10^–4^, *R*^2^ adjusted = 0.58) bilaterally, and one in right subgenual cingulate region (SCC, MNI 17, 27, −9 with *p* < 10^–3^, *R*^2^ adjusted = 0.42). Both do not survive a 5% FDR correction.

**Fig. 4:**
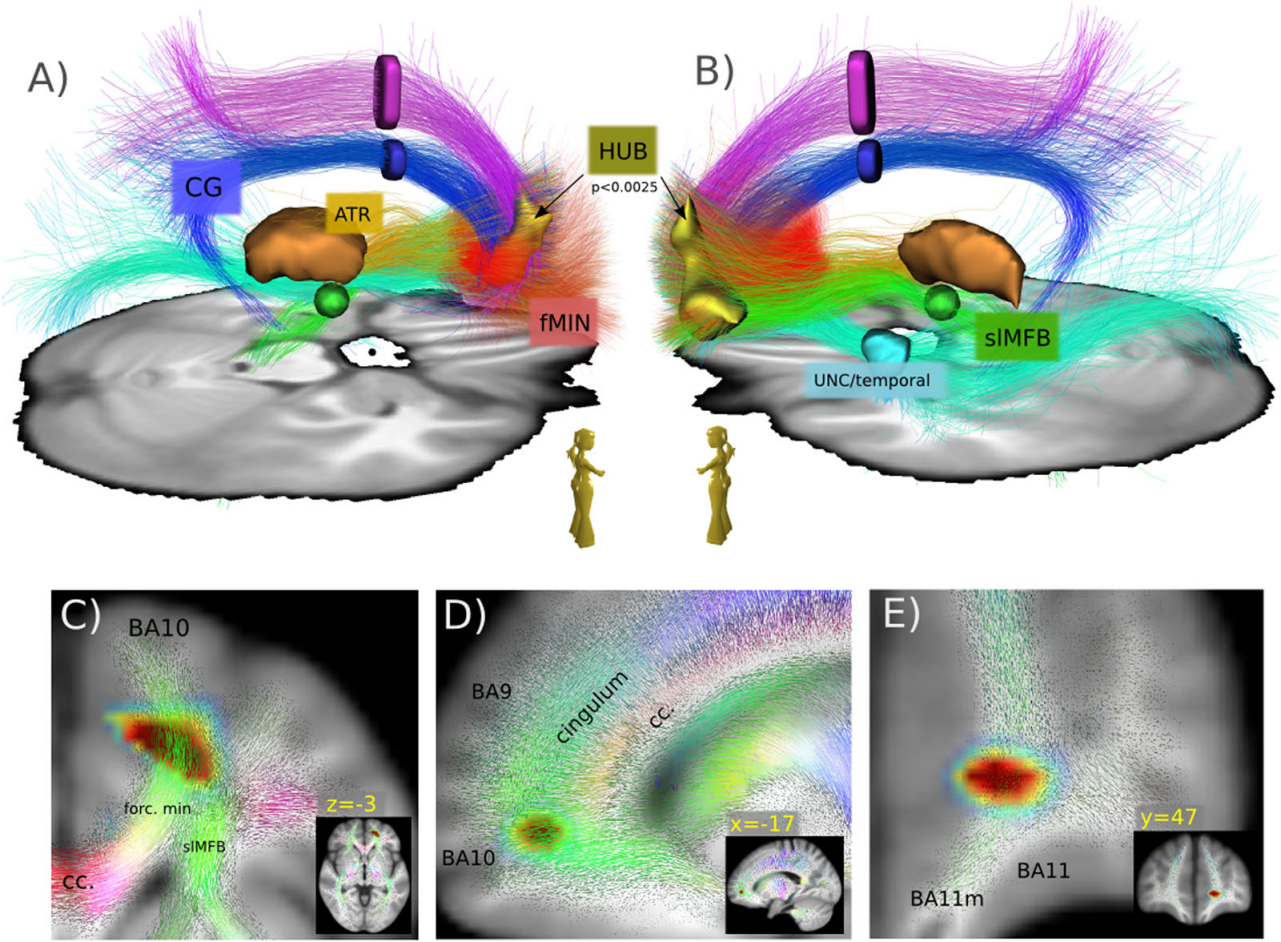
Qualitative depiction of the involved bundles. A qualitative view **a**, **b** of the major bundles involved in the HUB region. Tractography from a HCP group template, seeded from HUB (*p* < 0.01). Tracts are further separated (ROIs). Anterior thalamic radiation (ATR), superolateral medial forebrain bundle (slMFB), forceps minor (FMIN), cingulum (CG), uncinate fascicle (UNC), inferior fronto-occipital fascicle (IFOF), and superior anterior fascicle (SAF). **c**–**e** colored quiver plots give a prototypical impression of the local white matter geometry in the neighborhood of the HUB region

**Fig. 5:**
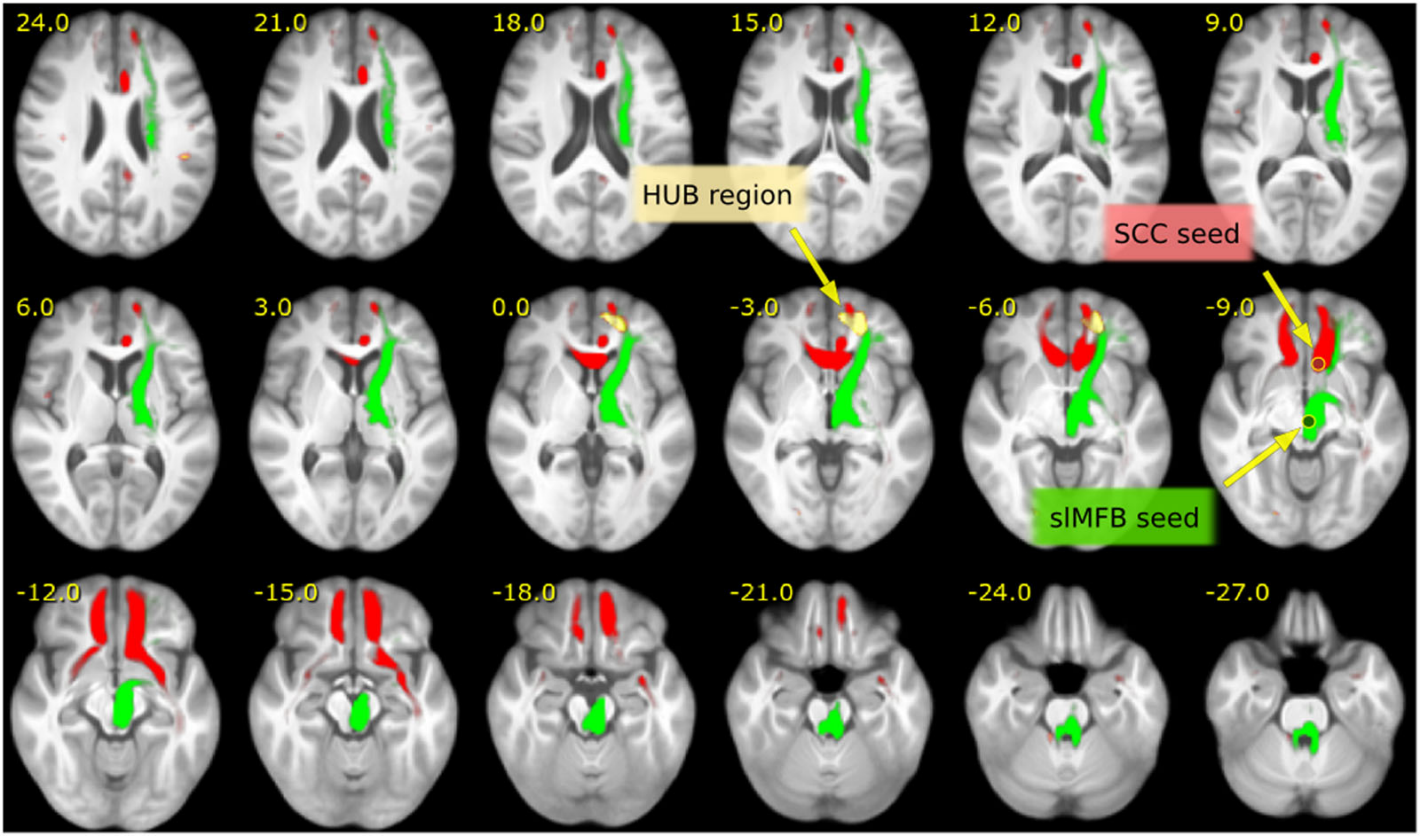
Comparison of the slMFB and SCC target. Comparison of networks seeded by the slMFB (green) stimulation target, (MNI: 6, −12, −8) and the SCC (red) target (MNI: 6, 22, −7.5) derived from Riva-Posse et al.^15^, Fig. 4. Both systems address the left frontal HUB region. Tractography was performed on the HCP group template and seeded in spherical regions (*r* < 3 mm) in the above given MNI coordinates

To understand the networks involved a whole-brain fiber reconstruction on an HCP group template was conducted and streamlines passing through the significant left fronto-polar region (at *p* < 0.01) were selected. In fact, all major fiber pathways (see Figs. 4a, b and 5) addressed before in effective DBS in MDD^15,16^ passed through the left fronto-polar region, which suggests that it constitutes a hitherto unknown branchpoint (HUB) of the emotional network.

The volume of this HUB region of the MDD group was analyzed in relationship with a group of healthy volunteers. No significant difference was found for the HUB volumes when comparing our total MDD group with the control sample. However, the non-responders (MADRS response <50%) were found to have a significant negative difference (*p* < 0.00001) from the control group. The slMFB DBS response hence seems to separate a subgroup from the clinical homogenous MDD group which is significantly different from healthy subjects.

To rate the quality of the HUB volume as an predictive biomarker a leave-one-out regression analysis was performed (see Fig. 2). If 50% reduction in MADRS is defined as the threshold for treatment response, 20 out of 24 subjects are correctly predicted as (non-)responders. For a better understanding of the effect strength: the size of a found region is ~5–7 ml (depending on the significance threshold), the relative volume changes within the considered cohort is ~15%. Thus, on individual level the volume changes are ~1 ml.

## Discussion

The presented analysis is complementary to the usual analysis via pathway activation modeling (PAM) based on VAT^14–16,28^, which in our cohort could not explain response variability. Electrode positions in the cohort were probably too uniform with respect to the targeted slMFB due to tractographic guidance of electrode implantation^18^. Moreover, VAT studies rely on certain simplifications, overestimate the size of the actual activated tissue volume^28^ and might not work in pure white matter stimulation ^18^.

Our finding suggests that response variability might originate from the existence of different phenotypes. Clinically, patients have a uniform symptom spectrum^8^. Within this group, white matter morphometry shows a certain imaging phenotype that is significantly correlated with response (volume expansion in left HUB, volume reduction in DLPFC and SCC right). Frontopolar alterations of microstructural FA in similar location have been described^29^. Also, reduced FA in connections of the VTA to dorsolateral frontal region have been discovered^30^, where mean FA was negatively correlated with depression scale rating scores. Other groups have found - albeit less significant - a volume reduction in the same frontopolar HUB region^31^ in MDD. This volume reduction could be the hallmark for the loss of connection to the midbrain (and can be interpreted as loss of connection to VTA as an important regulator of aversive and hedonic responses through slMFB, resulting in a midbrain volume reduction, cf. Fig. 2). HUB volume increase might coincide with full functional disconnection from the subcortical network (ATR, slMFB) which potentially leads to the previously described intrinsically high activity of the (left > right) frontal lobe^32,33^. In this sense MDD is seen as a continuum where ongoing disconnection is confluent with severity, and at a certain point implies treatment resistance. Whether slMFB DBS can change the HUB volume over time, lead to structural reorganization and a (functional) reconnection of the VTA to the dorsolateral frontal region is a question for future research.

Non-responders to DBS have previously failed non-invasive stimulation treatments (ECT)^8^. These non-responders contradictorily show increased volume in DLPFC potentially indicating a better connection of these superficial regions with the frontal network. In this respect it is not clear why a more focused technology like DBS does not work in this subgroup^34^. Thus, we suggest a distinct phenotype that precludes our MDD population from therapeutic non-invasive stimulation (and DBS), despite a presumed given network access over DLPFC in the non-responder subgroup. It has to be noted, however, that rTMS (repetitive transcranial magnetic stimulation) has not been tried on a regular basis in this cohort.

In conclusion, focal volume alterations might indicate activity changes in and disconnection from the main emotional network. A correlation of focal volume changes with response to slMFB DBS indicate a hidden feature (imaging phenotype) that cannot be identified on clinical grounds. VAT analysis in slMFB DBS shows similar fiber segment allocation in responders and non-responders, supporting optimal delivery of stimulation. Furthermore, slMFB DBS addresses the same network as HF stimulation of SCC (cf. Fig. 5). These results have direct clinical implications since for the first time biomarkers have been identified that might allow to identify future responders to DBS therapy, which would be a clear step into the direction of personalized treatments for psychiatric disorders. Whether the predictive power of the HUB volume change is enough to make reliable predictions on subject level is matter of future research.

### Limitations

Several limitations apply: a clinical DTI sequence (only 32 gradient directions) was used for implantation and post hoc analysis. The low resolution of this dMRI and the inherent inability of dMRI tractography to disentangle situations where neurites pass through a small bottleneck might have influenced the VAT based connectivity analysis. Thus, we cannot exclude, that there is an association with treatment response potentially measurable with scientific DTI data. For comparison with a normative sample we had to rely on the HCP sample (different scanners and acquisition protocols), because a true control group was missing. This does not narrow the significance of the within group effect, but makes the comparison with the normative sample questionable. At least, our MDD group was not distinguishable from the norm in the HUB region, which supports the validity of the comparison.

The left frontal HUB region showed a volume decrease in non-responders. This volume difference cannot be unequivocally attributed to any of the fiber tracts that traverse this region. Although we attributed this volume growth to the network of frontal white matter - and not the slMFB itself - the VTA/midbrain connection to the frontal lobe and its disconnection is important. An alternative explanation, however, could be that the slMFB in its head/pole region is volume increased itself.

## Supplementary information

Supplemental Material
