# Supplementary material for: Frontal white matter architecture predicts efficacy of deep brain stimulation in major depression
Source: Transl Psychiatry. 2019 Aug 21;9:197. doi: 10.1038/s41398-019-0540-4 (PMC6704187; doi:10.1038/s41398-019-0540-4)
Supplement: Supplementary file 1 — Supplemental Material [file 41398_2019_540_MOESM1_ESM.docx]

Supplemental Material

|  | Mean | SD | Minimum | Maximum | Median | Correlation with response (p) |
| --- | --- | --- | --- | --- | --- | --- |
| age | 48 | 11 | 29 | 71 | 48 | 0.54 |
| onset | 19 | 12 | 5 | 51 | 15 | 0.94 |
| MADRS | 39 | 6 | 15 | 39 | 30 | 0.85 |
| #episodes | 6 | 8.6 | 1 | 30 | 2 | 0.37 |
| MADRS response | 58 | 37 | 0 | 100 | 63 | --- |

A table describing statistics over the patient cohort with respect to patient age, onset times, MADRS response, the number of episodes and relative MADRS response to therapy. The significance level of correlations test of all variables with the response variable is given in the last column.
